# Cognitive processes of binomial expressions across modalities: a study of EFL Arab learners

**DOI:** 10.1007/s10339-026-01338-4

**Published:** 2026-04-22

**Authors:** Amjad Almudawi, Mohammed Mohsen

**Affiliations:** 1 Najran University, 662531 Najran, Saudi Arabia; 2 Qatar University, 664412 Doha, Qatar

**Keywords:** Binomial expressions, EFL learners, Formulaic language, Cognitive processing, Visual method, Auditory method

## Abstract

Processing binomial expressions (BEs) as a type of formulaic language remains an under-researched area in psycholinguistics despite extensive research on other forms of formulaic language, such as idioms, proverbs, phrasal verbs, and collocations. This study attempts to investigate how Arab learners of English as a Foreign Language (EFL) processed binomial expressions with different phrase types (congruent, incongruent, or both in their L1 and L2) over two different modalities: visual versus auditory modality. To this end, 70 intermediate EFL Arab participants took part in a treatment where they were exposed to three different phrases over two different modalities. Participants first rated their familiarity with congruent and novel binomials on a five-point scale and determined whether pairs of phrases were translation equivalents. Then, they were exposed to different BEs with either visual or auditory modality using SuperLab 6.0 software. Their processing of BEs was operationalized by reporting their reaction time, which was generated by the software. Results of their reaction time showed significant differences across modality and phrase type. Visual modality presentation resulted in accelerated processing times for all binomial expressions. Congruent phrases were processed most quickly in both modalities, which were followed by novel binomials in both languages and Arabic only. These findings suggest that visual presentation facilitates the quick processing of BEs regardless of phrase type.

## Introduction

Language is a complex system where words are often combined to form meaningful units that surpass their individual meanings. These combinations, which are referred to as multiword expressions (MWEs), are crucial in spoken and written communication, thus constituting a significant part of language usage. Recent studies (Green and Birdsong 2024; Du et al. 2021; Siyanova-Chanturia and Sonbul 2025) have highlighted the importance of broadening the research on bilingual language processing to include more than just individual words. This expansion should encompass lexical units, such as binomial expressions (BEs), which have been largely overlooked especially in the context of second language acquisition (SLA) and bilingual processing. BEs are pairs of words from the same lexical class that are joined together by a coordinating conjunction. They are commonly found in formulaic language. Mollin (2014) described them as “coordinated word pairs whose lexical elements share the same word class.” (p. 1). Examples such as “salt and pepper,” “knife and fork,” “bride and groom,” or “men and women” demonstrate how these expressions often have a conventional word order that is determined by various linguistic and social factors. Despite their prevalence, BEs have received relatively little attention in L2 research compared with other formulaic sequences, such as idioms or collocations. The importance of studying BEs stems from several factors. First, formulaic language, which includes binomials, accounts for a significant portion of spoken (58.6%) and written (52.3%) discourse in English (Erman and Warren 2000). This high frequency underscores the need for nonnative speakers to master these expressions for effective communication. Second, recent studies by Carrol et al. (2016) have shown that formulaic language, including binomials, enhances language processing efficiency by providing “ready-made frames” for expression and comprehension. This improvement can reduce cognitive load and support a fluent, native-like learner. Third, Arabic, as a heritage language, contains fixed formulaic expressions that are widely inherited from the Quran and frequently used in formal Arabic contexts, which can cause confusion when compared with daily spoken Arabic dialects. This feature necessitates studying formulaic language, particularly binomials, in Arabic and English to address the gap that learners face when encountering these expressions in real-life situations.

Traditional grammar does not include holistic representations of MWEs mainly because of the dominance of the linguistic theory known as the words-and-rules approach. According to this approach, the lexicon consists of memorized and stored forms, while grammar consists of rules that are applied to these forms (Ullman, 2001). Only morphemes, words, and highly distinctive forms, such as idioms, are believed to be part of the lexicon, excluding compositional language. Forms that can be created compositionally are typically not stored. Moreover, regular forms are created by adding suffixes each time they are used. However, one theoretical possibility is that some highly frequent regular forms may be stored, which is driven by memory limits on linguistic knowledge.

Despite the dominance of the words-and-rules approach, frequency-based approaches challenge the distinction between words and phrases or lexicon and grammar (Abbot-Smith & Tomasello, 2006) as well as the separation of lexicon and grammar. These approaches argue that our memory representations are shaped by our interactions with language forms, which consider language as a statistical accumulation of experiences that evolve with each encounter of linguistic units (Siyanova-Chanturia et al. 2011). Frequency effects are believed to apply to all language exemplars regardless of their length, complexity, abstractness, or literality, thus extending beyond words and idioms (Davis 1989).

The present study requires the frequency-based approach, where the frequency of BEs will be calculated according to the Sketch Engine Corpus and Google as a big corpus. Given that the frequency with which formulaic sequences appear is crucial to language learning, multiword sequence frequency has an impact on acquisition, representation, and processing (e.g., Bybee, 2013). The words-and-rules approach will not be the focal point in the study because the stimuli that have been selected in the experiment were extracted from the similarities and differences between L1 and L2.

While BEs are crucial in language use, research that tackles this area has been scant. According to Siyanova-Chanturia et al. (2011), binomials, despite being more prevalent than idioms, have not been thoroughly examined in SLA research from the perspective of psycholinguistics. This insufficient exploration has led to little knowledge on how L2 learners process these important linguistic elements in their mind given that these words are fixed and have different degrees of reversibility in terms of word order. Therefore, this study fills this gap by understanding how L2 learners mentally process BEs that were presented through two input modalities: visual and auditory. This study is motivated by multifaceted factors. First, as Alotaibi and Alotaibi (2015) emphasized, binomials are crucial for improving learners’ language competency, specifically oral proficiency in the target language, thus contributing to accuracy, appropriateness, and overall word understanding. Students of English as a Foreign Language (EFL) can use these linguistic elements to improve their oral proficiency to sound less literal and monotonous in their speech and become increasingly native-like. Second, from a psycholinguistic perspective, this study offers valuable insights into the cognitive processes that are involved in processing BEs. We employ online measures, such as translation recognition tasks, and analyze the correlation between accuracy and response time using SuperLab software. In doing so, we can gain a deep understanding of how Arab English learners navigate the similarities and differences in binomial phrases across different modes. Therefore, the main objective of this study is to understand how L2 learners process BEs across the different phrase types presented through two different modalities: visual versus auditory. Specifically, the research aims to answer the following question:

*How do different phrase types (novel in both languages, Arabic-only, and congruent) presented in two different modalities (visual vs. auditory) influence the processing of binomial expressions by Arab EFL learners?*

## Literature review

### Formulaic language and BEs

Formulaic language plays a crucial role in spoken and written communication, thus accounting for a significant portion of discourse across languages. Erman and Warren (2000) estimated that formulaic sequences comprise 58.6% of spoken discourse and 52.3% of written discourse in English. This prevalence underscores the importance of formulaic language in achieving native-like fluency and proficiency at the L2 level. Within this broad category of formulaic language, BEs represent a specific type that has received relatively less attention in research compared with other forms, such as idioms or collocations. BEs, as defined by Malkiel (1959: 113, cited from Millon, 2014), is “the sequence of two words pertaining to the same form-class, placed on an identical level of syntactic hierarchy, and ordinarily connected by some kind of lexical link.” Examples such as “bread and butter” or “right and duties” illustrate how these expressions often have a conventional word order that is determined by various linguistic and social factors.

The study of BEs is important in the Arabic context for several reasons. First, Arabic, as an inflectional language, differs significantly from English in its approach to formulaic language. As noted by Kaye (2009), Arabic binomials are often the result of a still-productive semantic category, whereas English binomials are typically used as frozen pairs. According to Kaye (2009), BEs in Arabic, unlike in English, tend to start with a positive word and followed by a negative one, such as “al-ḥayāh wa-al-mawt” (life and death), al-abyaḍ wa-al-aswad (white and black), and “bashīr wa-nadhīr” (bearer of good news and warner). The word order of these BEs also signifies cultural explanation especially in colors, such as “al-yad al-bayḍāʾ” (white hand), thus indicating generosity. Meanwhile, the expression “Ādam wa-Ḥawwāʾ” (“Adam and Eve”(, indicates that the male names come first in Arabic culture. Meanwhile, some BEs do not have identical counterparts in English, such as “al-akhḍar wa-al-yābis” (the green and the dry), thus opening a new path for investigation in research.

The acquisition and processing of BEs in L2 contexts involve complex interactions between various factors. Schmitt (2004) suggested that formulaic sequences, including binomials, can be acquired either holistically or incrementally. Wray (2002) argued that while young and adult native speakers tend to process language holistically, adult L2 learners typically process language analytically, which can potentially affect their fluency. Recent studies have provided evidence for the incidental acquisition of formulaic sequences from context. For instance, Durrant and Schmitt (2010) found that adult L2 learners retain information about lexical patterns to which they are exposed in input, thus supporting usage-based models of language acquisition. In terms of processing, several factors, such as congruency between L1 and L2, frequency of occurrence, semantic transparency, and L2 proficiency, have been identified as influential. Some studies (Carrol et al. 2016; Gyllstad and Wolter 2016) have shown that congruent items (L1 = L2) are typically processed more quickly than incongruent ones (L1 ≠ L2). In addition, Sonbul (2015) and (Wolter & Yamashita, 2018) demonstrated that high-frequency items tend to be processed efficiently by native speakers and L2 learners. Gyllstad and Wolter (2016) found that semantic transparency impacts processing with more transparent items being processed more easily than opaque ones. Additionally, Sonbul (2015) and Sonbul and El-Dakhs (2020) have shown that as L2 proficiency increases, learners become sensitive to certain factors, such as frequency and congruency in processing formulaic language. These findings highlight the multifaceted nature of binomial acquisition and processing in L2 contexts.

### Visual versus auditory modes

The impact of visual and auditory input modalities on the processing and acquisition of BEs is an emerging area of research in psycholinguistics. However, majority of studies (e.g., (Mohsen & Almudawis 2021; van Zeeland & Schmitt 2013; Vidal 2011) have focused on vocabulary learning outcomes, thus omitting the identification of how learning is processed through the mind. Recent studies have compared how different input modalities may affect the processing and acquisition of formulaic language. For instance, (Tuzcu 2023) observed that reading while listening demonstrated great success in promoting the attention to target words and led to the long-term and stable retention of novel words. Conklin et al. (2020) investigated the impact of simultaneous auditory input on reading patterns. Findings indicated that L2 speakers were often not synchronized with the audio when reading. Webb and Chang (2022) found that the reading-while-listening condition showed the greatest improvement in learning multiword combinations, which was followed by the listening-only and reading-only conditions. These studies suggest that visual input may have some advantages over auditory input in binomial processing and acquisition. However, the combination of both modalities may be the most effective. Additional research is needed to understand fully how different input modalities affect the processing and acquisition of BEs, particularly in L2 contexts.

### Relevant work

Congruency, which refers to whether a binomial has a translation equivalent in the learners’ first language (L1), has been the issue of several studies in L2 cognitive processing across different languages. For example, Chen and Fang (2024) found that Chinese EFL learners processed congruent binomials significantly faster and more accurately than incongruent ones with L1-lexicalized items showing further advantages. This congruency effect appears advantageous across studies in the Swedish context (Wolter & Gyllstad, 2013), which demonstrated that Swedish-English bilinguals processed congruent collocations faster than incongruent collocations with both subject to L2 frequency influence. Yamashita and Jiang (2010) found that Japanese-English bilinguals made few errors on congruent collocations regardless of proficiency. Arabic-English research contexts enhanced these patterns: Sonbul et al. (2025) found that students’ translators performed well in translating congruent phrases while their performance in incongruent expressions were at the below-than-expected levels. Altamimi (2024) showed Arabic nonnative speakers exhibited a rapid processing of congruent English collocations with effects modulated by proficiency. Meanwhile, El-Dakhs et al. (2020) examined Arab learners, which revealed complex interactions among congruency, task type, and proficiency. Theoretical accounts attribute congruency effects to early acquisition of shared forms, automatic cross-language coactivation, and reduced processing competition (Wolter & Yamashita 2017). These findings establish congruency as a fundamental psycholinguistic principle that affects bilingual MWE processing. However, the effects interact with proficiency and task demands.

Many recent studies have determined how Arab EFL learners process English BEs. For example, Alotaibi et al. (2022) examined the incidental learning of BEs (e.g., “wires and pipes”) by Arabic learners of English in three input modes: reading-only, listening-only, and reading-while-listening modes. The researchers also examined the effect of the frequency of exposure (2, 4, 5, and 6 occurrences) on learning. Participants read three stories in different modes. Each story contained novel and existing binomials. Two posttests assessed learners’ knowledge: a multiple-choice test and familiarity ratings. While findings indicated that no significant difference was observed between the reading-only and reading-while-listening groups, the two modes significantly outscored the listening-only group. The frequency of exposure had a significant effect on familiarity ratings but not on the multiple-choice test except when comparing the minimum (2) and maximum (6) exposures in the listening-only condition. Novel binomials that were seen six times became as familiar as existing ones. However, their form recognition remained low. The study showed that the incidental learning of binomials is possible through various input modes. Reading-based modes are more effective. Additionally, repeated exposure can incrementally build knowledge of new multiword sequences in a classroom context.

In a similar Arabic context, Sonbul and El-Dakhs (2020) examined how congruency (the availability of a direct L1 translation) and estimated L2 proficiency affect Arab EFL learners’ recognition of English collocations. The researchers conducted two experiments. Experiment 1 used an untimed multiple-choice test, while Experiment 2 employed a timed acceptability judgment task. Results showed that congruency had a substantial effect on untimed collocation recognition across all proficiency levels with participants scoring higher on congruent than incongruent collocations. However, in the timed task, the effect of congruency was modulated by proficiency level with the L1 influence diminishing as the estimated proficiency increased. The study also found that frequency effects were present in the timed processing for native and nonnative speakers, thus supporting usage-based models of language development. The authors suggest that these findings have implications for L2 teaching, testing, and research practices, thus emphasizing the need to consider timed and untimed measures when assessing collocation knowledge. They also note that the results challenge some aspects of the theoretical models of bilingual lexical processing, such as Jiang’s (2000) model of L2 lexical fossilization.

Regarding how Arabic native speakers process novel English binomials, Sonbul et al. (2023) investigated how nonnative speakers of English (L1 Arabic) process novel BEs in context. The researchers used eye-tracking to monitor participants’ reading of short stories that contained existing and novel binomials in different frequency conditions. The study addressed two main questions: whether nonnatives developed sensitivity to novel binomials in L2 input and what effect frequency of exposure had on this sensitivity. Results showed that nonnative speakers did not demonstrate a processing advantage for existing binomials over their reversed forms unlike native speakers. For novel binomials, nonnatives read subsequent encounters significantly faster than initial ones regardless of the frequency condition. Notably, the final reversed form resulted in accelerated reading times, thus indicating that L2 speakers treat the reversed form of a novel binomial as an additional instance by disregarding the original sequence. This outcome indicates that while nonnatives can quickly develop sensitivity to cooccurrence restrictions, they may not initially build sensitivity to the preferred word order. The study highlights the need for further research on the factors that influence the acquisition of BEs in L2 contexts.

In a non-Arabic context, Siyanova-Chanturia et al. (2011) studied how native and nonnative English speakers (N = 72) processed English idioms presented either literally, figuratively, or as novel in biased contexts using an eye-tracking program. The findings showed that native speakers processed target words faster with no significant differences among the three types of idioms. By contrast, nonnative speakers processed idioms at a similar level to novel ones but struggled significantly more with figurative idioms than literal ones. This outcome indicates that nonnative speakers require additional exposure to English idioms to improve their processing of novel and figurative expressions.

In a Chinese context, Du et al. (2021) examined how cross-language congruent words were processed by English-Chinese bilinguals, Chinese-English bilinguals, and English monolinguals. Participants viewed congruent, English-only, Chinese-only, and control binomials. Chinese-English bilinguals showed priming only for congruent items, thus reflecting L1 influence on L2. English-Chinese bilinguals exhibited a weak congruent effect but no priming for the other types, thus suggesting L1 inhibition in immersion. Monolinguals were primed for congruent and English-only binomials but not for Chinese-only ones. Overall, the results indicate bidirectional influence with a strong effect from L1 to L2.

### Research gap

Previous studies have extensively examined the processing of formulaic language by native and nonnative speakers by focusing on idioms, collocations, and binomials, and identifying several factors, such as L1–L2 congruency, frequency, semantic transparency, and L2 proficiency. However, a significant gap remains in our understanding of how learners process BEs across different modalities. Most research has employed visual presentation modes with a limited exploration of auditory input. Moreover, studies that compare input modalities have largely concentrated on single words. The present study aims to address these gaps by investigating how learners process L1 and L2 congruent and incongruent binomials presented through visual and auditory modes. Reaction times were recorded using SuperLab 6.0. This research expands on previous works by incorporating three types of BEs: congruent, novel in both languages, and Arabic-only. It also builds upon the existing/novel categorization of Sonbul et al. (2025). The novel aspect of this study is the use of SuperLab 6.0 to track students’ processes of BEs, which is done by recording their reaction time during exposure to BEs in visual and auditory modes. The aim is to gain an improved understanding of how different sensory inputs can affect the processing of BEs in L1 and L2.

## Methodology

### Research design

This study employed a quasi-experimental design with a quantitative approach to investigate the processing of BEs among Arab EFL learners. The research examined how different categories of binomials (congruent, novel in both languages, and Arabic-only) were processed across visual and auditory modalities. The quasi-experimental nature of the study stemmed from the nonrandom selection of the sample with comparisons drawn between two distinct groups: participants using visual modality and those using auditory modality. The quantitative approach entailed the collection and analysis of numerical data to measure variables and assess relationships between them objectively. This methodology incorporated descriptive statistics (including mean, standard deviation, and variance) and inferential statistics (such as t-tests and analysis of variance) to provide a comprehensive analysis of the data.

Two primary measures were employed in this study:

1. SuperLab 6.0: Used to measure participants’ response times and accuracy as they process congruent and incongruent binomial phrases between Arabic and English.
2. Online Questionnaire: Designed on a five-point scale to assess participants’ familiarity with existing and novel binomials.

Two translation recognition tasks, which were termed translation acceptability judgment tasks, were administered on campus using laptops: one through visual modality and the other through auditory modality. These tasks were designed to test participants’ ability to decide whether two phrases presented to them sequentially represented the same meaning or were translation equivalents.

### Participant selection

The study involved 70 female Saudi EFL university students majoring in English and Translation at the College of Languages and Translation (blind for peer review purpose). Participants were recruited as a convenience sample from different educational levels. They were categorized as intermediate level as per the standardized test performed by the department, which corresponded to levels B1 and B2 on the Common European Framework of Reference for Languages (*M* = 4.39, *SD* = 0.32) and B1 in the IELTS. All participants shared similar backgrounds in terms of native language (Arabic), nationality (Saudi), age, (M = 21.2, SD = 1.02) culture, and educational background. They were familiar with various translation techniques and possessed basic computer skills that were necessary for the study. We selected the participants based on their availability and willingness to participate. Those who agreed to take part in the study were asked to sign a consent letter.

### Materials

We were interested in selecting the BEs which had the same linear order of the words either in Arabic or English to prevent confusion and interference in cultural matters. The study material consisted of BEs that were categorized into three groups based on their similarities and differences in Arabic and English:

1. Congruent: BEs that exist and have the same direction in Arabic and English. For example, “Food and drink” (طعام وشراب in Arabic), “cause and effect” (سبب ونتيجة in Arabic).
2. Novel in both languages: BEs that are nonexistent in either language. For instance, “Chickens and rabbits” (دجاجات وأرانب in Arabic), “wires and pipes” (أسلاك وأنابيب in Arabic).
3. Arabic-only: BEs that exist in Arabic but not in English. For example, “ساحر أو مجنون” (Magician or madman in English) and “خِصم وحَكم” (opponent and judgment in English).

The selection of BEs was based on a review of relevant literature, including *Arabic-English-Arabic-English Translation: Issues and Strategies* by Husni and Newman (2015) and “First Language Influence on the Processing of Formulaic Language in a Second Language” by Conklin and Carrol (2018). Another criterion for selecting the BEs was the high frequency of these words based on the Sketch Engine Corpus and Google as a large corpus. Notably, the frequency was based on the Modern Standard Arabic (MSA), which is a variety of Arabic that is widely used only in formal contexts, such as media, formal writing, and formal education, and categorized by some scholars (Ibrahim & Aharon-Peretz 2005) as a second language. The participants of the current study speak a Saudi dialect that is not identical to the MSA.

After setting the BEs from Arabic sources, they were translated into English by the authors and checked for appropriateness and equivalence by two professional translators. One was an associate professor of translation, while the other was a freelancer translator. In total, the experiment included 30 BEs and 30 fillers. The 30 binomial phrases were equally divided into the three types described above (10 congruent phrases, 10 novel phrases in both, and 10 Arabic-only phrases). In each modality (visual and auditory), participants encountered 5 congruent phrases, 5 novel phrases in both, 5 Arabic-only phrases, and 15 fillers (See Appendix A).

#### Binomials and their phrase frequency

The frequency of occurrence for all phrases was determined based on the Sketch Engine Corpus and Google as a large corpus. Phrases were categorized into three groups based on their recurrence: high, mid, and low. All phrases with low or medium frequency were classified as novel in both languages or Arabic-only, while phrases with high frequency were classified as congruent. The words that composed these phrases were selected to have high frequencies to ensure appropriateness for the participants’ level and avoid confusion or ambiguity.

#### Fillers

Thirty phrases were included as filler items. They were grammatically correct phrases with the same syntactic structure as binomials but lacked plausibility, such as “chapter and picture” and “early and global.” Half of the fillers were used in the visual test, while the remaining half in the auditory test. The Arabic translations of the fillers differed from their English equivalents in the two tests.

### Research instruments

#### Online questionnaire

The online questionnaire was created using Google Forms (https://www.google.com/intl/ar/forms/about/). This platform was selected based on its successful use in previous studies and its ease of use for creating surveys and analyzing data. The questionnaire was used to conduct a familiarity rating task prior to the treatment, where participants rated their familiarity with the BEs on a five-point scale in their native language (4 – highly familiar to 0 – not familiar at all). The questionnaire’s initial draft was peer-reviewed by a panel of applied linguistics and linguistics experts to assess statement appropriateness, thus resulting in numerous modifications to ensure reliability and face validity before administration. Cronbach’s alpha was 0.84, thus indicating a good reliability of the questionnaire.

#### SuperLab 6.0

**Fig. 1:**
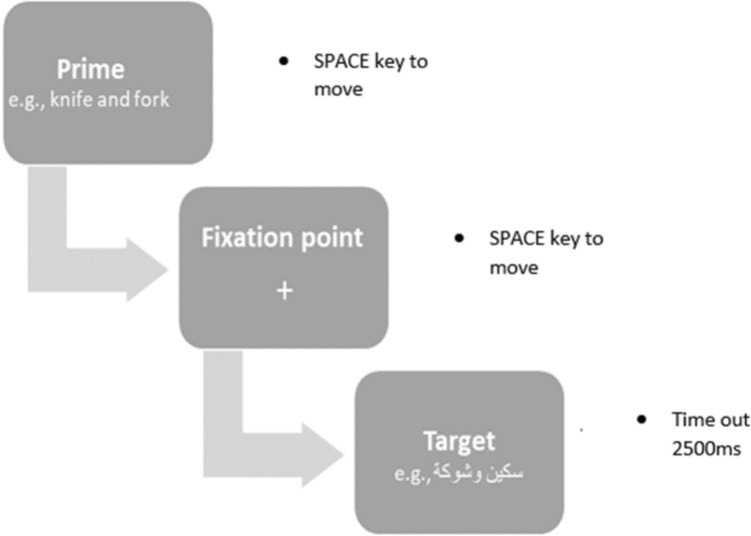
Translation acceptability judgment task (Task 1)

**Fig. 2:**
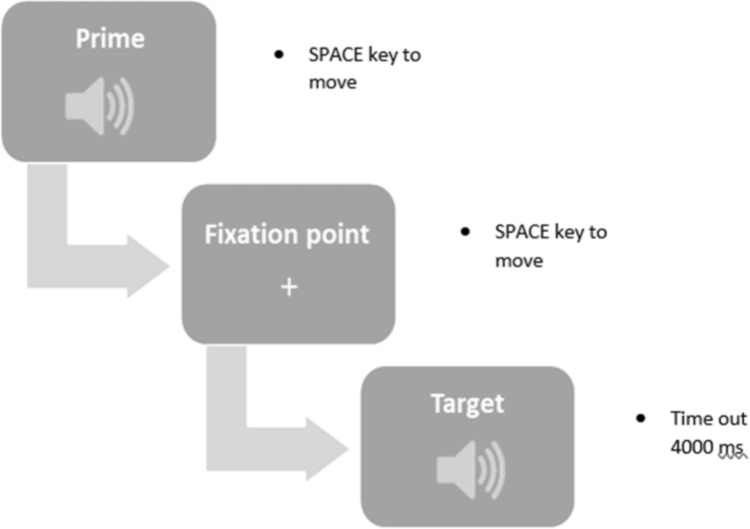
Translation acceptability judgment task (Task 2)

SuperLab is a stimulus presentation software that allows for the design of experiments that require visual stimuli on screen, audio stimuli via speakers, and control or synchronization with other devices. It records responses and reaction times in a text-only file that can be read by most spreadsheet or statistics programs. SuperLab is compatible with Mac OS X and Windows operating systems and used as a measurement tool to test 1) response time in milliseconds, where 1000 ms equal 1 s) and 2) accuracy, that is, whether they answer correctly or not). It was used to conduct two translation acceptability judgment tasks (the first through visual modality and the second through auditory modality). Screenshots from the software were provided to explain to the student how they ran the two tasks (See Figs. 1, 2).

SuperLab performs two main functions: (1) Script Editor, which is used to create new experiments or tests, and (2) Runtime Program, which is used to run the experiment and save response information in a file. In SuperLab, responses are recorded on the keyboard or on external devices connected to a computer. The software allows for customized stimulus presentation. The order of stimuli is divided into blocks of trials with each consisting of a series of events or stimuli. Stimulus duration can be customized within the constraints of the video display refresh cycle. The reaction time is calculated as the time between the beginning of the stimulus and the response.

### Pilot study

A pilot study was conducted with 12 participants who were not included in the main study. The purpose of the pilot study was to examine the feasibility of the SuperLab 6.0 software and the suitability of Google Forms in collecting data. The goal was to familiarize students with the experiment tools, address any potential challenges or questions that students may have, and assess the appropriateness of the materials and the students’ level of understanding. All participants were selected from the same section as the original sample and shared identical characteristics regarding language proficiency, age, and cultural background. During the pilot study, some technical issues were identified and addressed. For example, participants without Google accounts were provided alternative ways to answer the online questionnaire. Additionally, the time limit for the primes (English phrases) in the SuperLab tests was found to be too short, thus causing anxiety and stress for some participants. These issues were addressed in the main study design. The results of the pilot study showed that participants could use the required software, SuperLab 6.0, and that the item selection criteria were suitable and could be used in the main study sufficiently.

### Procedure

The study first adhered to ethical commitments by obtaining ethical approval (reference No.: 009610-020891-DS) IRB committee at (anonymized for peer review). Consent forms were obtained from all participants before the experiment began. Participants who agreed to take part were added to a WhatsApp group created by the first author. A video explaining the research methodology, including instructions on how to answer the online questionnaire and use SuperLab software, was shared with the group. A schedule for campus attendance to perform the translation recognition tasks was also provided.

**Fig. 3:**
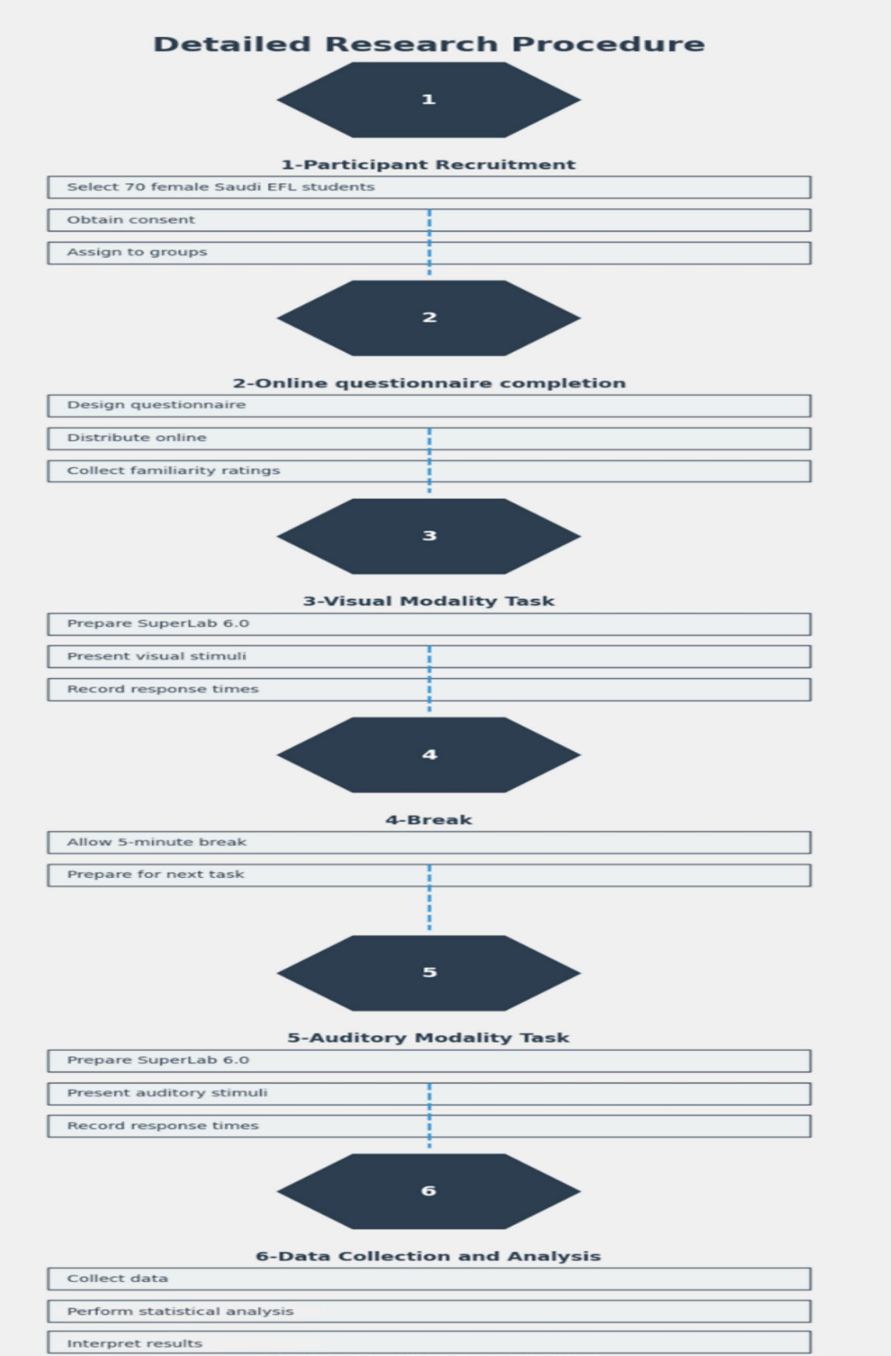
Visualization of the study procedure

The procedure for the main study began with participants completing the online questionnaire for familiarity ratings prior to performing the translation recognition tasks. Using SuperLab 6.0, participants completed two translation acceptability judgment tasks. The first was a Visual Modality Task, where instructions were presented on the computer screen followed by 30 practice trials. Each trial consisted of a fixation point (“ + ”) presented in the center of the screen, which was followed by an English phrase prime (e.g., “knife and fork”) that remained until a response was made or the item timed out at 2,500 ms. The fixation point then reappeared, after which the Arabic phrase target (e.g., "سكين وشوكة") was displayed until a response was made. After a five-minute break, participants began the Auditory Modality Task, which followed a similar procedure to the visual task but with all items presented auditorily and a timeout of 4,000 ms per item. In both tasks, participants used the keyboard to indicate whether the phrase was correct (“x” key) or incorrect (“z” key). All items were presented in black lowercase letters using Times New Roman (Headings CS) font and size 24 pt over a gray background. Data from participants with an error rate of 30% or more were excluded from the analysis. For those with less than 30% errors, only correct responses were included. The entire experiment took approximately 20 to 30 min per participant with no incentives provided because of the relatively short time commitment. The detailed procedure was summarized in the following infographic (see Fig. 3).

### Data analysis

We answered the research questions by employing a multistep analytical approach. First, we assessed the normality of our data distribution by calculating skewness and kurtosis values, which fell within the normal range (− 1 to + 1). Reaction times to the visual and auditory modes generated from the SuperLab software were computed and analyzed to operationalize students’ processes to the target BEs. We also utilized descriptive statistics to compute means and standard deviations of students’ responses to the online questionnaire to check their familiarity with the BEs. For comparative analysis, we applied inferential statistics, namely, a two-way analysis of variance (ANOVA), to examine differences in students’ processing between the two presentation modes. All statistical analyses were conducted using the R software package, thus ensuring robust and reliable results throughout our study.

## Results

**Fig. 4:**
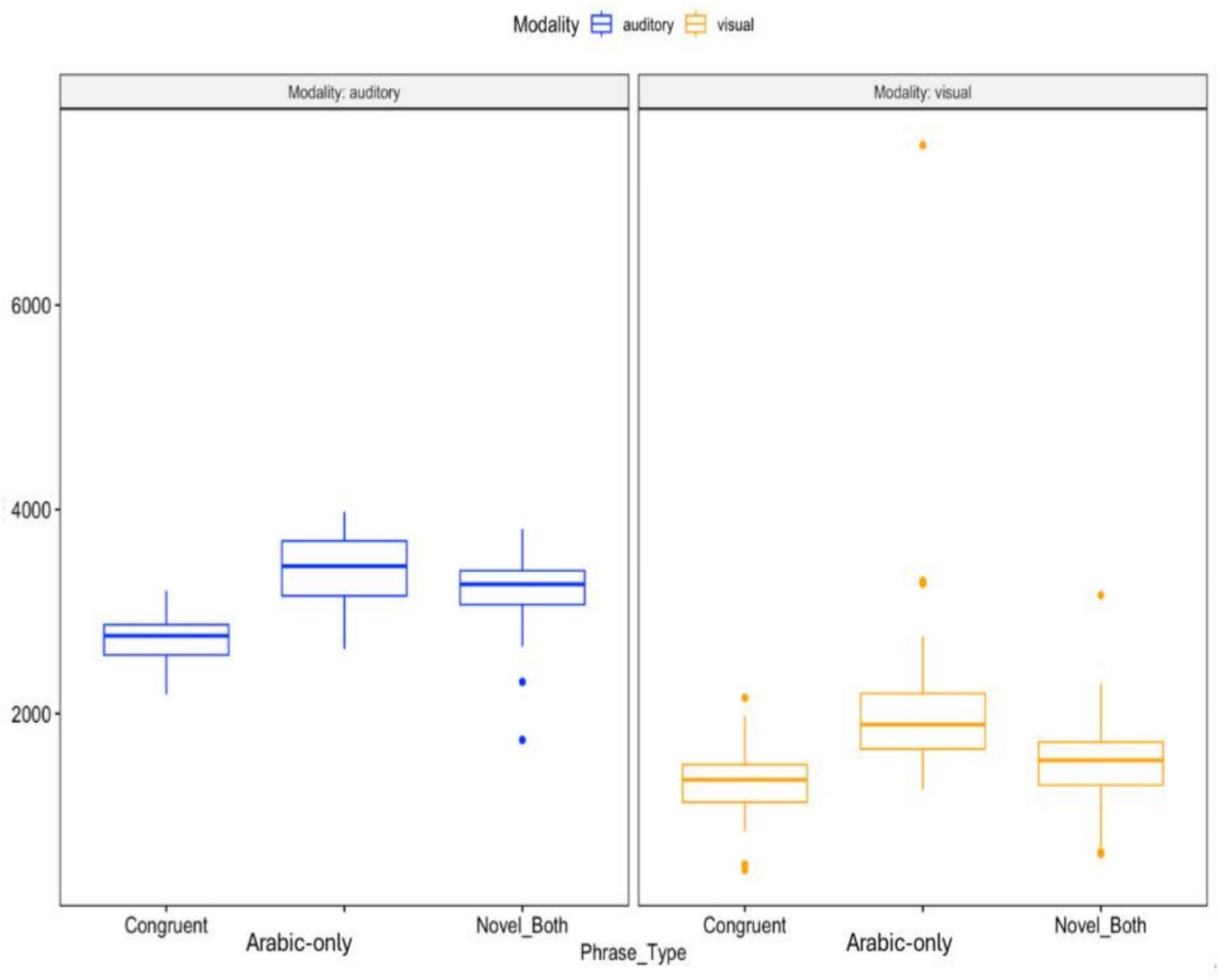
Averages of phrase types across different modalities

- Reaction times.
  The reaction times (RT) in milliseconds (msec) of the participants were analyzed in R using two-way (2 × 3) repeated measures ANOVA with two independent variables. The first is phrase type with three levels (congruent, Arabic-only, and novel in Arabic and English). The second variable is modality with two levels (auditory and visual) with the alpha level at 0.05. Descriptive statistics were first used to calculate the reaction times across phrase type over two modes, which were summarized in Table 1. Inferential statistics are also placed in Table 2.
  The results are presented in Table 2. Figure 4 shows that a significant difference was obtained for the main effects of modality (F(1, 362) = 1008.1, p < 0.001, η2 = 0.65) and phrase type (F(2, 362) = 71.8, p < 0.001, η2 = 0.09)). In addition, a significant interaction of modality and phrase type was observed F(2, 362) = 6.47, p = 0.0017, η2 = 0.008).
  A post-hoc pairwise comparison was conducted to assess the significant interaction between modality and phrase type. A pairwise comparison using Tukey’s test was performed to determine significant differences in accuracy (correct, error, and no response) across visual modality. The results of this comparison are summarized in Table 3.
  The pairwise comparison using Tukey’s test revealed significant differences among all response types in the visual modality. Correct responses were most frequent, which was followed by errors and then no responses. This outcome was evidenced by significant differences between error and no response (p = 0.001), error and correct (p < 0.001), and no response and correct (p = 0.005). The test statistics indicated that errors occurred more than no responses (0.833), correct responses were more common than errors (− 1.567), and correct responses were more frequent than no responses (− 0.733). These results suggest that in the visual modality, participants were more likely to attempt an answer, with a higher tendency towards correct responses, rather than not responding at all.
  Similarly, for the auditory modality, a pairwise comparison using Tukey’s test was performed to determine significant differences in accuracy (correct, error, and no response) across students’ responses. The results are summarized in Table 4, which indicates a significant difference among responses.
  The pairwise comparison using Tukey’s test showed significant differences between most response types in the auditory modality. Errors occurred significantly more often than no responses (p < 0.001, test statistic = 0.900), while correct responses were significantly more frequent than errors (p < 0.001, test statistic =  − 1.400). However, the difference between no responses and correct responses was not statistically significant (p = 0.053, test statistic =  − 0.500) but approached significance. These results suggest that in the auditory modality, participants were most likely to give correct responses or make errors with few instances of no response. The lack of significant difference between no responses and correct responses indicates that participants were almost equally likely to provide a correct answer or no answer when presented with auditory stimuli.
- Google forms experiment.
  The questionnaire, which is known as the Familiarity Rating Task, was administered to assess the participants’ familiarity with the binomials examined in the present study. A Google Forms link was created featuring a five-point Likert scale to facilitate participation. The participants rated their familiarity with existing and novel binomials (1 = I have never heard or used this phrase, 2 = I have very rarely heard or used this phrase, 3 = I have rarely heard or used this phrase, 4 = I have frequently heard or used this phrase, and 5 = I have very frequently heard or used this phrase) prior to participating in the reaction time experiment. Four categories were identified for the Arabic translations used in the data analysis: (a) most familiar items, (b) familiar items, (c) undefined items, and (d) unfamiliar items.
  The questionnaire results are presented in Appendix B. Items were ranked from highest to lowest based on familiarity ratings. The most familiar item was “brother and sister” (*M* = 4.75, *SD* = 0.82), which was followed by “sunrise and sunset” (*M* = 4.74, *SD* = 0.81) and “sun and moon” (*M* = 4.72, *SD* = 0.83). According to the participants’ responses, these items were considered the most familiar because their mean scores were close to 5. Several phrases had mean scores around 4, thus indicating general familiarity among participants. Examples included “knife and fork” (*M* = 4.48, *SD* = 1.09), “food and drink” (*M* = 4.24, *SD* = 1.26), and “cause and effect” (*M* = 4.07, *SD* = 1.49). Some phrases had mean scores around 3, thus suggesting that while the majority of students were familiar with them, a notable portion might not have understood these expressions. Examples included “stories and jokes” (*M* = 3.40, *SD* = 1.27), “bags and coats” (*M* = 3.31, *SD* = 1.45), and “opponent and judgment” (*M* = 3.20, *SD* = 1.46). Additionally, four phrases had mean scores around 2, thus indicating that they were unfamiliar to most participants: “sword and guest” (*M* = 2.40, *SD* = 1.43), “fathom or cubit” (*M* = 2.21, *SD* = 1.47), “captivity and camel” (*M* = 2.15, *SD* = 1.38), and “goats and pigs” (*M* = 1.97, *SD* = 1.29).

**Table 1:** Mean of each modality across different phrase types

Modality	Phrase type	Reaction time (in milliseconds (mesc.)
Auditory	Congruent	2727.5
Visual	Congruent	1294.61433
Auditory	Arabic-only	3400.19718
Visual	Arabic-only	2159.52459
Auditory	Novel both	3245.7047
Visual	Novel both	1472.07224

**Table 2:** Effects of modality

Response: RT					
	Sum Sq	Df	F value	p-value	Effect size (η2)
Modality	205053952	1	1008.1389	< 0. 002*	0.65
Phrase_Type	29225160	2	71.8421	< 0. 002*	0.09
Modality x Phrase_Type	2635396	2	6.4784	0.001*	0.008
Residuals	73630263	362			

*Significant

**Table 3:** Pairwise comparison of total responses for visual phrases

Sample 1–sample 2	Test statistic	Std. error	Std. test statistic	Sig
Error-no response	0.833	0.258	3.227	0.001*
Error-correct	− 1.567	0.258	−6.068	0.000*
No response-correct	− 0.733	0.258	−2.840	0.005*

*Statistically significant at 0.05

**Table 4:** Pairwise comparison of total responses for auditory phrases

Sample 1–Sample 2	Test statistic	Std. error	Std. test statistic	Sig
Error-no response	0.900	0.258	3.486	0.000*
Error-correct	−1.400	0.258	− 5.422	0.000*
No response-correct	−0.500	0.258	− 1.936	0.053

*Statistically significant at 0.05

The results indicate that RTs were significantly faster in the visual modality than in the auditory modality across all phrase types (p < 0.001). Regardless of the phrase type, participants responded more quickly when presented with visual stimuli (overall visual modality mean = 1575.42 ms) than with auditory stimuli (overall auditory modality mean = 3085.26 ms). In terms of pairwise comparisons among different phrase types, the RT for congruent phrases was significantly faster than that of novel Arabic phrases in the auditory modality (p < 0.001). Additionally, the RT for congruent phrases was significantly faster than that of the novel in both phrases type (p < 0.001). However, no significant difference in RT was observed between the novel Arabic phrase type and the novel in both phrases type in the auditory modality (p = 0.08). For the phrase types in the visual modality, a significantly faster RT was observed for congruent phrases than that for novel Arabic phrases in the visual modality (p = 0.019). Similarly, the RT for congruent phrases was significantly faster than that of the novel in both phrases type (p < 0.001). Additionally, the RT for the novel in both phrases type was significantly faster than that of the novel Arabic phrase type in the visual modality (p < 0.001). In addition, the questionnaire analysis aimed to determine whether different levels of familiarity with BEs differentially influenced RT based on the similarities and differences between Arabic and English. The major findings of this analysis confirmed that the similarities and differences between the two languages affected familiarity ratings. This result aligns with the recognition task findings, which also showed an effect of congruent and incongruent binomials.

## Discussion

This study aimed to investigate the processing of BEs by Saudi EFL learners across visual and auditory modalities. The results reveal several key findings that contribute to our understanding of L2 binomial processing and expand upon previous research in the field.

### Visual vs. auditory processing

The study found significantly faster RTs in the visual modality than in the auditory modality across all phrase types. This outcome aligns with previous research that suggested that visual input may have advantages over auditory input in L2 processing (Tuzcu 2023; Webb & Chang 2022). Our findings support those of Alotaibi et al. (2022), who discovered that the reading-only and reading-while-listening conditions resulted in better performance than the listening-only condition in the incidental learning of BEs. This visual advantage could be attributed to several factors, including the holistic nature of orthographic processing compared with the sequential nature of phonological processing (Rosenzweig and Bennett 1996) and participants’ strong familiarity with written English because of their academic background. Additionally, the visual presentation is fixed, thus allowing participants to refer back to the stimulus as needed. By contrast, auditory presentation is transient and fleeting, thus becoming impossible for listeners to revisit the content, which may increase cognitive load (second author, 2016). Our study aligns with findings by Author and a colleague (2021) who reported that students need to be exposed to auditory input three times to reach the same level of L2 vocabulary acquisition as achieved through a single exposure to visual input. Another factor could be that orthographic processing may be more holistic than the sequential nature of phonological processing, thus leading to an accelerated input to high-level processing systems. This holistic approach allows for the simultaneous processing of all elements in a visual stimulus, thereby potentially reducing cognitive load compared with the sequential unfolding of auditory stimuli (Second author, 2016). Additionally, the participants’ strong familiarity with written English, given their academic background, may have contributed to this effect. This outcome is particularly relevant in the Saudi context, where exposure to spoken English is limited and English education often emphasizes written language. Therefore, students may build strategies that develop robust processing strategies for written input. Additionally, the nature of the recognition task itself may have favored visual processing because visual presentation allows participants to refer back to the stimulus if needed, whereas auditory presentation is transient and relies heavily on working memory. However, our results contrast with Vidal’s (2011) findings, which suggested that listening was as effective as reading for learning. A plausible explanation for this discrepancy is that our study’s focus was different from Vidal’s concentration. While Vidal’s research was product based and examined learning outcomes, our study was process based and focused on the real-time cognitive processing of binomial expressions.

### Congruency effects

Our findings revealed a fascinating pattern: congruent BEs were processed more swiftly than novel BEs in visual and auditory modalities. This observation adds weight to the growing body of evidence supporting the facilitative effect of L1–L2 congruency in formulaic language processing (Wolter and Gyllstad, 2013; Carrol et al. 2016). It is as if our participants’ brains were saying, “Hey, I recognize this pattern from my native language!” and processing it more efficiently as a result. This phenomenon aligns with Sonbul and El-Dakhs’s (2020) findings, where congruency played a substantial role in untimed collocation recognition across various proficiency levels. The results also corroborate with the line of research findings that reveal that processing congruent BEs is easier than incongruent phrases (Altamimi 2024; Chen and Fang 2024; Yamashita and Jiang 2010). Our results indicate that congruency is not only significant in processing but also surpasses incongruency in terms of translating BEs from and into L1 and L2. This outcome is in line with Sonobol et al.’s (2025) findings, which revealed that L1 and L2 translation is more difficult for incongruent phrases because of the difficulty in processing these phrases in the students’ mind. The persistence of this congruency effect at intermediate proficiency levels is intriguing. It suggests that our L1 knowledge continues to be a silent partner in our L2 processing even as we become increasingly proficient. This observation lends support to Schmitt’s (2004) proposal that formulaic sequences can be acquired holistically and incrementally. In the case of congruent BEs, our L1 knowledge provides a ready-made template, thereby potentially facilitating a holistic acquisition process.

### Novel BE processing

In the visual modality, an unexpected finding emerged: novel BEs in both languages were processed faster than those that were novel only in Arabic. This result suggests that completely unfamiliar phrases may be processed differently from those with partial L1 familiarity, which is a phenomenon that merits further investigation. Our participants’ exposure to BEs classified as novel in Arabic may be limited despite Arabic being their native language. This limitation is partly due to the linguistic gap between colloquial Arabic and MSA because the latter is used in formal written contexts. Some researchers, including our second author (2021), have proposed that MSA may be considered an L2 for Arab learners. This linguistic divide may explain why participants struggled to process certain unfamiliar MSA binomial expressions.

Our findings extend the work of Sonbul et al. (2023), who observed that nonative speakers quickly developed sensitivity to cooccurrence patterns in novel binomials but not to the preferred word order. Our results indicate that even native Arabic speakers may face similar challenges with formal written language. When encountering a BE that is novel in both languages, learners may process it as an entirely new unit, thus potentially avoiding L1 interference. Conversely, BEs that are novel only in Arabic may activate partial L1 knowledge, thus resulting in a competition between L1 and L2 representations and slow processing. This interpretation aligns with the concept of cross-linguistic influence in L2 processing (Kroll and Stewart 1994) but suggests a more complex interaction than previously understood.

### Familiarity ratings and their influence on RT

Our results indicated high familiarity among students with congruent binomials, as reflected by the high mean scores in the familiarity rating task. Notably, the development of binomial knowledge observed in this task mirrored that in the form recognition task, thus demonstrating a clear sensitivity to similarities between Arabic and English binomials. For instance, the questionnaire results showed that the most familiar items were “brother and sister,” “sunrise and sunset,” and “sun and moon.” These phrases represent the most familiar items in the questionnaire because they are among the most common examples of congruent binomials between Arabic and English.

Furthermore, several other phrases were reported to be familiar to participants but to a lesser extent. Examples include “knife and fork,” “food and drink,” and “cause and effect,” which also fall within the category of congruent binomials but with slightly lower familiarity ratings. By contrast, some phrases, such as “stories and jokes,” “bags and coats,” and “opponent and judgment,” were less defined for participants because certain expressions are not shared between both languages, while others exist only in Arabic. Finally, four phrases, namely, “sword and guest,” “fathom or cubit,” “captivity and camel,” and “goats and pigs,” appeared unfamiliar to participants. These expressions are primarily used in Arabic and rarely encountered in English, which likely led participants to rate them as unfamiliar.

The results support usage-based models of language acquisition (Durrant and Schmitt 2010) by demonstrating that learners can quickly develop sensitivity to novel BEs through exposure. However, the congruency effect and the differences in processing between types of novel BEs suggest that L1 influence remains strong, thus challenging aspects of Jiang’s (2021) model of L2 lexical fossilization. Our findings extend the work of Wray (2002) on the holistic versus analytical processing of formulaic language by L2 learners. While Wray suggested that adult L2 learners typically process language analytically, our results indicate that congruent BEs may be processed holistically, thereby suggesting a more flexible approach to L2 processing than previously thought. The study also contributes to our understanding of how input modality affects BE processing, which is an area that has previously been underexplored in the literature. The clear advantage of visual processing across all BE types suggests that modality plays a crucial role in L2 formulaic language processing, thus extending beyond the single-word level investigated in previous studies.

## Conclusion and suggestions for future research

This study investigated how Saudi EFL learners process BEs in visual and auditory modalities. Our findings reveal that visual presentation of BEs leads to faster processing than auditory presentation. Additionally, congruent BEs are processed more quickly than novel ones. BEs that were new in both languages were processed faster than those that were only new in English, thus suggesting that partial familiarity might actually slow down processing. These results highlight the complex relationship between a learner’s first and second languages in processing formulaic language and underscore the importance of considering different input modes in language learning and teaching. However, our study has several limitations. It focused only on Saudi female university students, which limits the generalizability of our findings. We examined immediate processing rather than long-term learning. As such, we cannot draw conclusions about language acquisition over time. Given that this study’s focus did not target phrase frequency, future studies can investigate this variable to support our study’s findings. Individual differences among participants and the artificial nature of the experimental setting were not fully taken into account. Future research can address these limitations and expand our understanding by including a diverse group of participants and exploring BE processing changes over time through longitudinal studies. Investigating how individual differences affect BE processing and learning, examining BE processing in naturalistic settings, and exploring the neural mechanisms underlying this process using brain imaging techniques can provide valuable insights. Additionally, studies on how different types of instruction affect BE processing and learning, as well as comparative studies between different language pairs, can further our understanding of cross-linguistic influence in BE processing. These future directions aim to build upon the current study and deepen our knowledge of how learners process and acquire BEs in a second language, thus potentially informing effective language teaching strategies.

## Data Availability

Data will be provided upon request with convincing reasons.

## Appendix B. The results of questionnaire

**Table:**

	Not at all familiar		Unfamiliar		Undefined		Familiar		Very familiar		Mean	Std. deviation
	N	%	N	%	N	%	N	%	N	%		
Brother and sister	2	2.9	1	1.4	2	2.9	2	2.9	63	90.0	4.75	0.82
Sunrise and sunset	2	2.9	0	0	4	5.7	2	2.9	62	88.6	4.74	0.810
Sun and moon	2	2.9	1	1.4	2	2.9	4	5.7	61	87.1	4.72	0.83
Knife and fork	3	4.3	3	4.3	5	7.1	5	7.1	54	77.1	4.48	1.08
Food and drink	5	7.1	4	5.7	7	10.0	7	10.0	47	67.1	4.24	1.26
Cause and effect	11	15.7	1	1.4	6	8.6	6	8.6	46	65.7	4.07	1.497
Shelves and drawers	3	4.3	9	12.9	11	15.7	7	10.0	40	57.1	4.02	1.28
Name and address	7	10.0	8	11.4	7	10.0	7	10.0	41	58.6	3.95	1.43
Time and money	6	8.6	5	7.1	14	20.0	10	14.3	35	50.0	3.90	1.33
Magician or madman	7	10.0	5	7.1	14	20.0	8	11.4	36	51.4	3.87	1.38
sorrows and wakefulness	7	10.0	6	8.6	14	20.0	9	12.9	34	48.6	3.81	1.38
Bottles and tins	6	8.6	9	12.9	13	18.6	10	14.3	32	45.7	3.75	1.37
Theory and practice	4	5.7	7	10.0	19	27.1	16	22.9	24	34.3	3.70	1.20
Games and music	4	5.7	13	18.6	14	20.0	9	12.9	30	42.9	3.6	1.34
Ladies and gentlemen,	14	20.0	6	8.6	8	11.4	3	4.3	39	55.7	3.67	1.65
Paint and glue	10	14.3	7	10.0	13	18.6	11	15.7	29	41.4	3.60	1.46
Stories and jokes	6	8.6	10	14.3	24	34.3	10	14.3	20	28.6	3.40	1.278
Bags and coats	10	14.3	12	17.1	17	24.3	8	11.4	23	32.9	3.31	1.45
opponent and judgment	14	20.0	7	10.0	19	27.1	11	15.7	19	27.1	3.20	1.46
Stone and wood	11	15.7	16	22.9	15	21.4	5	7.1	23	32.9	3.18	1.49
Kin and strangers	17	24.3	10	14.3	18	25.7	4	5.7	21	30.0	3.02	1.55
Bull and ewe	24	34.3	5	7.1	11	15.7	9	12.9	21	30.0	2.97	1.67
Wires and pipes	18	25.7	10	14.3	20	28.6	6	8.6	16	22.9	2.88	1.47
Chickens and rabbits	21	30.0	13	18.6	12	17.1	6	8.6	18	25.7	2.81	1.58
Boyhood and fun	22	31.4	11	15.7	15	21.4	13	18.6	9	12.9	2.65	1.42
Days and eras	25	35.7	11	15.7	14	20.0	7	10.0	13	18.6	2.60	1.51
Sword and guest	27	38.6	14	20.0	13	18.6	6	8.6	10	14.3	2.40	1.43
Fathom or cubit	35	50.0	9	12.9	12	17.1	4	5.7	10	14.3	2.21	1.47
Captivity and camel	33	47.1	13	18.6	13	18.6	2	2.9	9	12.9	2.15	1.38
Goats and pigs	41	58.6	4	5.7	15	21.4	6	8.6	4	5.7	1.97	1.29
